# How an EPA-based curriculum supports professional identity formation

**DOI:** 10.1186/s12909-022-03116-0

**Published:** 2022-01-20

**Authors:** Anne E. Bremer, Marjolein H. J. van de Pol, Roland F. J. M. Laan, Cornelia R. M. G. Fluit

**Affiliations:** 1 Department of Radboudumc Health Academy, Postbus 9101, 6500 HB Nijmegen, The Netherlands; 2 Department of Primary and Community Care, Radboudumc, Nijmegen, the Netherlands

**Keywords:** Entrustable professional activities, Feedback, Participation, Professional identity formation, Undergraduate medical education

## Abstract

**Background:**

Entrustable professional activities (EPAs) are widely used in medical education, and they might be an important incentive to stimulate professional identity formation (PIF) of medical students, by actively encouraging participation in the workplace. The goal of this study was to explore the effects of an EPA-based curriculum on the PIF of medical students in undergraduate curricula.

**Methods:**

In this study at the Radboud University Medical Center in Nijmegen, the Netherlands, the authors interviewed twenty-one medical students in three focus group interviews (November 2019), and conducted a thematic analysis based on both the synthesizing concepts PIF, communities of practice and EPAs, and newly defined themes.

**Results:**

Four central themes proved crucial for understanding the influence of EPAs on PIF: creating learning opportunities, managing feedback, dealing with supervision in context and developing confidence**.** EPAs helped students to create learning opportunities and to choose activities purposefully, and the use of EPAs stimulated their feedback-seeking behavior. The context and way of supervision had a great impact on their development, where some contexts offer better learning opportunities than others. EPAs helped them develop trust and self-confidence, but trust from supervisors hardly appears to result from using EPAs.

**Conclusions:**

An EPA-based curriculum does stimulate PIF in the complex context of working and learning by supporting participation in the workplace and by encouraging feedback-seeking behavior. Striking the right balance between participation, feedback-seeking behavior and choosing learning activities is essential.

**Trial registration:**

This study was approved by the ethics committee of the Netherlands Association of Medical Education (NVMO, case number 2019.5.12).

**Supplementary Information:**

The online version contains supplementary material available at 10.1186/s12909-022-03116-0.

## Background

The professional behavior of healthcare professionals is essential as it is closely related to healthcare improvement [1]. The opposite is also true: unprofessional behavior by healthcare professionals can negatively impact healthcare teams and patients [2], and it can even lead to harming patients [3]. Medical schools, therefore, highly value their students’ professional behavior and aim to prevent unprofessional behavior by stimulating the professional identity formation (PIF) of their students. This has been a key concept in the development of medical education in the twenty-first century [4].

When entering medical school, students have already formed part of their personal identity shaping their personalities through life experiences [5]. Next to their personal identity, however, students also develop a professional identity, as shown by Cruess and colleagues [5]. Identity is developed through three domains; an individual identity (beliefs about yourself, personality), a relational identity (impact of significant others) and a collective identity (influence of social groups), all influenced by medical education [5]. Early participation in the clinical workplace can help students to take responsibility for their own role [6] and to develop a professional identity [7] and, in addition, it also encourages them to become more sensitive towards patients, which improves healthcare [8]. Professional identity formation (PIF) describes this process by which medical students develop the knowledge and skills they need to show professional behavior, and how they form such a professional identity [9]. PIF aims to ensure that learners understand and ultimately internalize the professional morals and ethics of their profession [10–12].

For PIF, the process of becoming a medical professional within the medical community is of great importance [13]. This community can be considered a ‘community of practice’. Communities of practice are “groups of people who share a concern or a passion for something they do and learn how to do it better as they interact regularly” [14]. Social interaction between the participants of the community of practice promotes learning. When medical students start out as newcomers, they will be on the outside or periphery of the community (legitimate peripheral participation) [5], but through social contact, experience, socialization and medical education, students will move towards more or (nearly) full participation in the medical department [5]. Ultimately, with full participation, they will have acquired the professional identity that is related to their community [5].

Entrustable professional activities (EPAs) are tasks entrusted to a learner as soon as sufficient skills are demonstrated to allow for unsupervised practice [15]. They are “… essentially units of significant clinical work” [16]. EPAs have been developed for graduate medical education [17] on which most studies concentrate [18, 19], but they are used more and more often in undergraduate medical education [20], on which this study focuses. In contrast to competencies, which are broadly defined personal abilities, EPAs represent specific tasks that have to be performed in the workplace. As EPAs describe activities that medical students are allowed and expected to perform during their clerkships [21], they are a way to transform competencies into specific formalized tasks for clinical practice [22] and they are used to stimulate participation of medical students in their clerkships.

In the use of EPAs in medical education, entrustment plays a key role. Medical students will be able to develop their competencies and work on their PIF process if they are actively supported by professionals in the workplace. Furthermore, the teaching environment and the interaction between medical students and other medical professionals is vital for student participation and development [8]. With appropriate trust, they find it easier to learn and contribute to their team, and to feel free to ask questions and show insecurities [23]. Besides, appropriate trust benefits students’ patient care [23]. In this way, it seems possible that entrustment and interaction with these significant others impact the development of the students’ relational identity.

In communities-of-practice theory, EPAs can be considered “practices”, that is, shared resources that guide learning and participation in the community of practice. We hypothesize that when students perform EPA-related activities, they will become more entrusted to perform them independently, and that, with entrustment increasing, they will participate more in the community and eventually become part of it. In this way, EPAs are the practices that make participation of medical students in the medical community possible, which might influence the development of their collective identity.

An EPA-based undergraduate curriculum was implemented in the Radboud University Medical Center in Nijmegen, the Netherlands, in 2019. We expect that EPAs can be an important incentive to stimulate professional development and PIF when they actively encourage participation. Since students themselves are responsible for the acquisition of sufficient feedback, they are expected to actively participate in the medical department. In this study we focus on EPAs instead of competencies, as EPAs specifically describe tasks to be performed in the workplace. However, not much is known yet about how the students’ PIF can be studied [24] or about the relation between an EPA-based curriculum and PIF. More knowledge of the relation between EPAs and PIF can increase the strength and the wider implementation of EPAs in undergraduate curricula.

The goal of this study, therefore, is to explore the effect of an EPA-based curriculum on the professional development and the PIF of medical students. These findings will allow researchers and curriculum developers to improve the organization of their undergraduate medical programs, to guide their students’ PIF and to identify potential obstacles in the development of medical students.

## Methods

In this study we used a constructivist approach searching for the participants’ reality and their ideas about certain phenomena [25]. We acknowledge that our own background assumptions might have affected the research process, so we paid attention to reflexivity and how our own beliefs and expectations shaped the interpretation of the data. The lead author (AB) is an educationalist, CF is a professor in workplace learning with a medical and educationalist background, MP is an associate professor on wellbeing, a general practitioner and educational researcher and RL is a director of an educational institute, a professor in medical education and MD.

### Setting

The setting of this study was the renewed undergraduate medical curriculum at the Radboud University Medical Center in Nijmegen, the Netherlands, where medical students spend 3 years of clinical rotations in different hospital and general practice settings. The curriculum consists of five different EPAs, all including sub-EPAs: [1] medical consultation, [2] medical procedures, [3] guidance and education, [4] communication and collaboration and [5] non-clinical activities. For an example of one of the EPAs with related sub-EPAs see Table 1, and an overview of all EPAs can be found in Additional file 1: Appendix A.

**Table 1:** Example of an EPA

EPA 1: Medical consultation	
1.1 Anamnesis and physical examination	
1.2 Formulate differential diagnosis	
1.3 Formulate plan of investigation	
1.4 Interpret results of common diagnostic tests	
1.5 Formulate treatment plan	

Medical students are required to request feedback from their supervisors and other staff on a daily basis, at least 35 times in a 4-week clerkship. Supervisors have to provide narrative retrospective feedback along with a prospective entrustment-supervision scale grade on a specific EPA-related task. These grades are rather meant to be a feedback instrument than a formal assessment tool. All feedback reports are collected in a personal e-portfolio, which provides an overview of the activities students are entrusted to perform and their professional development. EPAs are used as a curriculum framework rather than a formal entrustment and assessment tool, though all the acquired feedback does serve as input for entrustment decisions.

All of the included medical students were in one of their first-year clerkships at the Radboud University Medical Center. The clerkships in year 1 last 4 to 8 weeks and are in a fixed order, and they take place at the university hospital and affiliated hospitals. Out of all undergraduate medical students, we chose first-year interns because this allowed us to collect relatively new information and fresh opinions about their experiences with the curriculum as they had only just embarked on their clerkships, and had not yet been fully integrated into the workings of the medical system. These students had already gained considerable experience with practice-oriented education, such as handling patient contacts and learning and working with an e-portfolio in the first 3 years of their curriculum.

### Study design

We adopted a purposive sampling strategy to select the participants. The first researcher (AB) contacted two groups of 30 interns via a briefing in a regular educational meeting. Out of these 60 students, 21 agreed to participate. In total, we held three focus group interviews with interns in their second and third clerkships.

A semi-structured interview guide was used in all three focus group interviews. This guide was built on different synthesizing concepts: *participation* and *communities of practice* [5], and *entrustment*, *feedback* and *entrustable professional activities* [21–23, 26]. These concepts were used to make sure the most important subjects were included, but the input of additional information was highly appreciated and stimulated during the focus group interviews. An experienced focus group interviewer moderated the interviews and AB and a research assistant (JT), CF and/or MP were present at every interview to take field notes. All interviews took place in a private space at the hospital. We completed our data collection when we came to conceptual depth [27] and reached our research goal.

### Data analysis

The interviews were audio-recorded and transcribed verbatim by AB and JT immediately after they had taken place. All data was anonymized and analyzed by thematic analysis by AB, CF and MP. We used a phenomenological approach; the experiences and perceptions of the participants were the main focus of this study. Initial synthesizing concepts were first defined by AB by deductive coding, based on literature about PIF, communities of practice and EPAs. CF and MP then coded the interviews for these initial themes.

Subsequently, the three researchers discussed the deductively developed themes and formulated new themes by inductive coding. Themes were updated until agreement was achieved, and a final code book was agreed by all researchers in a meeting prior to the final analysis. All three interviews were then coded to specific codes for the different themes using Atlas.ti (version 1.5.4), a qualitative research coding program.

## Results

Our respondents pointed out that EPAs contributed to their development in general and PIF in particular. EPAs made it possible for both students and supervisors to enhance their understanding of the students’ development, especially when they were surveyed over time. We determined four central and inter-related themes that help understanding the influence of EPAs on PIF: creating learning opportunities, managing feedback, dealing with supervision in context and developing confidence**.** In our EPA-based curriculum, these themes affected the students’ PIF. The results are supported by quotes, which have been indicated in the text with a ‘Q’ following the number of the quote, and can be found both in full text (the most relevant ones) as in Additional file 1: Appendix B (all quotes).

### Creating learning opportunities

Our respondents indicated that EPAs helped them to create learning opportunities and to choose activities purposefully (Q1, Q2). In addition, EPAs described important activities that students had to accomplish in a particular clerkship, and they gave them a clear overview of these activities. However, not all important activities appeared to be described, and students sometimes struggled with the need to observe their supervisor/preceptor and the need to practice themselves (Q3).

Besides guiding learning activities, EPAs help students to create boundaries for their learning activities. They explained that they needed to feel they had enough space and a sense of autonomy to develop, and EPAs provided natural boundaries to this (Q4). Lastly, EPAs showed students which activities they had to undertake in order to develop further:

*“It’s teaching you lessons all the time, things to improve or to do differently next time … In that sense, it helps you to know what you’re doing and what you’ll do again next time. When you’re getting negative feedback, you’re not going to incorporate that into your professional attitude. But you will do so the other way around: when you look back on something you did really well or when you were friendly or erm eager, you’ll show that more often. It’s a learning process.”* (Q5)

### Managing feedback

Our respondents reported that the proper functioning of the EPA system required them to ask for regular feedback, and that the use of EPAs stimulated their feedback-seeking behavior. They needed to ask for feedback often, and this created a prearranged moment for them to sit down with their supervisor, to reflect on the activity and to receive feedback, which they agreed was instructive. However, students mentioned that there was a risk of quantity being put before quality in the feedback they received, and they often expressed their concern of not receiving enough feedback and thus not achieving the expected EPA supervision levels:

*“And so you’re under a lot of pressure, with so many EPAs to obtain. It’s overstepping the mark, I think, actually you just want to get some feedback, which is very instructive and appropriate, but now there are so many EPAs that you worry about their number. That’s a bit of a shame, I think.”* (Q6).

Another point for attention was the feedback procedure, with students mentioning that they often filled out the feedback form based on the oral feedback they received before handing it to their supervisor. In this case, students first reflected on their own behavior and subsequently verified this with their supervisor. While this may help to strengthen the students’ reflective power, it may also facilitate a layback approach in the supervisors, preventing them from substantiating and complementing their feedback (Q7).

Besides the formal EPA feedback, our respondents also referred to the informal feedback they received in the workplace, e.g., during a patient visit or at the coffee machine. They greatly appreciated informal feedback, but they struggled with its inclusion in their e-portfolios as it was not always directly related to an EPA (Q8).

### Dealing with supervision in the context

The context and the way our respondents dealt with supervision appeared to have had a great impact on their development. There are considerable differences between hospitals, departments and doctors, and some contexts offer better learning opportunities than others, e.g. when they are more familiar with the EPA based curriculum or provide more or better supervision (Q9, Q10). Another important aspect of supervision is continuity in student guidance. Our respondents’ views differed about the best method of supervision, with some preferring being guided by one supervisor, and others by multiple supervisors (Q11, Q12).

*“I thought it was useful for someone to monitor my development; in the intermediate review talk, she told me what points needed attention. At the end, she saw the improvements I’d made throughout the process, so yes. I also think it’s useful to be getting occasional feedback from someone else. I happened to have two supervisors, so that was great, the other one noticed different things that he thought were important. But I valued that there was someone who saw the whole thing.”* (Q11)

Moreover, not only the supervision method has an impact on the medical students’ development, but also the duration of the clerkship itself and the impact of “starting again” every time (Q13, Q14). Furthermore, they explained that they felt supported and safe in a department where the staff knew their name, where they could actually contribute to the existing duties and where they felt free to ask for help. They appreciated being seen by the staff, and they highly valued being part of the team.

### Developing confidence

Trust plays a major role in developing confidence and in the students’ professional development. The students distinguished two different kinds of trust: trust in themselves (self-confidence) and trust from their supervisors. Firstly, they explained that EPAs helped them develop self-confidence:

*“When you’ve attained a particular EPA, you’re thinking ‘Wow, I’ve mastered it, and I can do this every time now’, something like that. In any case I think it helps to build your self-confidence, so you feel you can do it, and so I can actually be a doctor sometime.”* (Q15)

They also indicated, however, that they got this feeling of confidence not only from the EPA-based feedback but also from receiving informal feedback (Q16) and from informal experiences of success (Q17).

Secondly, entrustment from supervisors is also of great importance. Students can earn such entrustment, on the one hand, by showing their skills and capabilities and by taking initiative to act independently and, on the other, by asking for help in time. Trust from supervisors is particularly earned by performing activities in informal settings, but it hardly ever appears to result, however, from using EPAs and e-portfolios:

*“You do get more and more responsibilities but not on the basis of the EPAs you’ve attained. So there’s ongoing assessment of what they expect from you at that level and at that moment. In surgery, they just expect you to be able to do such and such. No one ever checks your EPAs except the final assessor or the intermediate assessor.”* (Q18).

Trusting medical students, moreover, greatly depends on the context: the level of cohesion and the degree of confidence amongst the staff in a medical department influence how much and how easily trust is given to students. However, EPAs can contribute to building relationships and creating learning opportunities for students and supervisors (Q19).

Furthermore, students develop confidence by using the narrative feedback and the prospective entrustment-supervision scale grades to show their experience to other supervisors:

*“It’s very valuable that, through EPAs, all the feedback is documented in your portfolio, so you can show another supervisor what you have done before.” (Q20)*

## Discussion

Our new and most important finding is that our EPA-based curriculum does stimulate PIF in the complex context of working and learning by striking a balance in participation in the workplace and feedback-seeking behavior. Because of the required number of feedback reports, EPAs stimulate the participation and feedback-seeking behavior of medical students, and they activate their learning process, which stimulates PIF. Our themes ‘Managing feedback’ and ‘Dealing with supervision in the context’ show that knowing how to manage feedback seeking behavior and how to deal with supervision, influence the development of the student’s relational identity [5]. EPAs represent the practices that encourage participation in the workplace, which enables students to move from legitimate peripheral participation to greater participation in their community of practice [5]. With this greater participation in the workplace, students develop their collective identity [5]. This is reflected in our themes ‘Creating learning opportunities’ and ‘Developing confidence’: EPAs support students in determining learning opportunities that allow them to participate in the medical department, which make them become increasingly confident and entrusted by the collective, the community of practice. Furthermore, EPAs assist supervisors in providing students with more and valuable feedback. The use of EPAs in the curriculum and the great amount of feedback related to the EPAs contribute to medical students’ intrinsic motivation and their lifelong learning attitude.

### Balancing EPAs and PIF

Our study showed that EPAs stimulate formal feedback, which is in line with previous research [28], but for optimal development, students must strike a balance between formal and informal feedback. Moreover, our finding that feedback-seeking behavior comes with the risk of quantity being put before quality is supported by the literature [29], stressing the importance of striking a balance between asking and over-asking for feedback. Furthermore, earlier research showed that EPAs support autonomy and act as motivators for important activities [30], and our findings support this view. However, students need to harmonize these activities with activities they personally prefer and consider useful for their development.

EPAs support the participation of medical students in the workplace. For PIF it is essential that the amount of both participation and autonomy are in balance [7]. Too much responsibility and autonomy can give rise to feelings of insecurity, so students need to have enough trust to ask for help in time. Our finding that appropriate trust helps students to learn, to contribute to their team, to feel free to ask questions and to show insecurities, is supported by the literature [23].

Finally, EPAs stimulate the supervisors’ engagement in the students’ PIF because of the feedback they are required to provide. Apart from the feedback itself, EPAs stimulate social interaction between students and supervisors, both members of the community of practice, which promotes learning and development [31]. It is vital for students’ PIF, however, to balance between the right amount of supervision and different supervisors. We showed that medical students benefit from receiving feedback from multiple perspectives, which underlines the importance of having more supervisors. However, they also highly value having a single supervisor who follows and guides their development, which stresses the importance of receiving feedback from the same supervisor for the opportunity of follow-up supervision, as supported by previous research [32].

### How non-EPA-related aspects support PIF

The above-mentioned results show that our EPA-based undergraduate curriculum supports the PIF of medical students. Besides formal feedback derived from EPAs, students also value informal feedback, which is supported by research showing that medical students consider informal feedback as highly valuable and very useful [33]. PIF is supported by the feedback students receive from early participation in the workplace during clerkships [7]. Not only people can provide feedback to students, but the different tasks in the workplace also function as feedback on their own. PIF is also stimulated by the non-EPA clinical responsibilities medical students are given [7] on a daily basis. Though all above-mentioned aspects are not directly related to the use of EPAs, indirectly they are, because an EPA-based undergraduate curriculum provides the right conditions for stimulating greater participation, feedback and responsibility.

### Entrustment

Our results show that students do not seem to be actively entrusted based on EPAs and portfolios, although they do show their former experiences and feedback as ‘evidence’ to supervisors. This could be explained by the implicit power of EPAs; entrustment decisions may be motivated by EPAs, but this might hardly be noticeable for students. Another explanation is the block rotation structure of the curriculum; students are naturally entrusted as soon as they pass through the clerkship and continue with the next. This implies that supervisors of a next clerkship assume that an acceptable amount of EPAs has been achieved at the end of a previous clerkship which makes them sufficiently entrusted to move on to the next. However, students seem not to be aware of this as they do not link this implicit entrustment with explicit entrustment decisions.

### Strengths and limitations

This study has two major strengths. The first of these is the responsive approach of the medical students and their willingness to explain their experiences. The focus groups consisted of students who had been working and studying together in the same group for at least 6 months, so they knew each other well, which created a safe atmosphere. They demonstrated a great eagerness to talk in-depth about their experiences and to cooperate with us aiming to improve the curriculum. In addition, the participants showed their willingness to learn from each other during the focus groups, which is the second strength of this research.

We acknowledge, on the other hand, that this study also has some limitations. The focus group interviews were held at one institution with its own unique organizational curriculum and culture, which might make this study less transferable to the population at large. Another limitation is the fact that our participants are diligent students, since they are in the first groups of the new master curriculum. They might be highly aware of their own professional development which could influence the results of this study. However, this study was a first exploration of how medical students purposely use EPAs for their PIF, and the perspective of these particular students has given us a very valuable first insight into how medical students develop.

### Implications for future research

When aiming to improve the use of EPAs in undergraduate curricula, we believe that two aspects are in need of further research. Firstly, we need to gain a better understanding of why (explicit) entrustment decisions based on EPAs seems to be underexposed by supervisors, while EPAs are developed for entrustment decisions. Secondly, it would be valuable to perform further research into how medical students perceive the transitions between clerkships and how they can be made to feel more comfortable at the start of a new clerkship. When medical students feel safe and part of the team, they find it easier to develop and participate in the workplace, which stimulates their PIF.

## Conclusions

Our EPA-based curriculum stimulates PIF by supporting more participation in the workplace and by encouraging feedback-seeking behavior. It is all about striking balances in participation, feedback-seeking behavior and choosing learning activities.

## Supplementary Information

**Additional file 1 Appendix A.** EPAs in the Radboudumc. **Appendix B**. Quotes by medical students about how their professional identity formation was affected by the use of entrustable professional activities.

## Data Availability

The datasets used and analysed during the current study are available from the corresponding author on reasonable request.

## Abbreviations

AB: Anne Bremer (first author)
CF: Cornelia Fluit (author)
e.g: Exempli gratia
EPA: Entrustable professional activity
JT: Jasmijn Terlouw (research assistant)
MP: Marjolein van de Pol (author)
PIF: Professional identity formation
RL: Roland Laan (author)
Q: Quote
