# Supplementary material for: How an EPA-based curriculum supports professional identity formation
Source: BMC Med Educ. 2022 Jan 20;22:48. doi: 10.1186/s12909-022-03116-0 (PMC8781044; doi:10.1186/s12909-022-03116-0)
Supplement: Supplementary file 1 — Additional file 1 Appendix A. EPAs in the Radboudumc. Appendix B. Quotes by medical students about how their professional identity formation was affected by the use of entrustable professional activities. [file 12909_2022_3116_MOESM1_ESM.docx]

**APPENDICES**

## **APPENDIX A: EPAs in the Radboudumc**

| **EPA** | **Sub-EPA** |
| --- | --- |
| 1. Medical consultation | - 1. Anamnesis and physical examination   2. Formulate differential diagnosis   3. Formulate plan of investigation   4. Interpret results of common diagnostic tests   5. Formulate treatment plan |
| 1. Medical procedures | 2.1 Participation in the operating room  2.2 Rectal examination, insert catheter  2.3 Common profession specific activities  2.4 Installing a drip, venous puncture, injections  2.5 Basic life support, Automated External Defibrillator |
| 1. Guidance and education | 3.1 Discuss diagnostic and therapeutic possibilities  3.2 Discuss results and prognosis  3.3 Difficult or unusual conversations  3.4 Provide information about a healthy lifestyle and prevention of diseases  3.5 Motivating interviewing |
| 1. Communication and collaboration | 4.1 Present oral and written reports that document a clinical encounter  4.2 Inter- and intraprofessional collaboration |
| 1. Non-clinical activities | This is not a clearly defined EPA; students can record any non-clinical activity |

**APPENDIX B: Quotes by medical students about how their professional identity formation was affected by the use of entrustable professional activities**

| **Quote number** | **Full quote** |
| --- | --- |
| Q1 | *“It helps to make your work more goal-oriented, I think.”* |
| Q2 | *“Well, when you’re on the ward and you’re thinking ‘right, I still need an EPA for physical examination’, you will try and do a physical examination that very same afternoon. When you need history taking, you’ll take a history.”* |
| Q3 | *“I was thinking that there are also things that don’t earn you EPAs but that are interesting in any case, so that’s the other side of the story…And so perhaps you’re less inclined to do those when the reward system is so clear-cut, but then again, you can’t do everything. Suppose you observe a procedure and it’s very interesting and really important to watch it being done, well, you’re not going to make something up in retrospect to get it assessed. So you spend time on it and that needs to be compensated some other time, and so it’s important not to raise the bar too high, causing people to skip things that are actually instructive because they’re chasing after their EPA quota.”* |
| Q4 | *“There’s a limit, of course. I mean, suppose you can do so many things that it’s making you insecure about your ability to do them, that would be uncomfortable. You could discuss this issue, of course. But erm I prefer having the option of being involved rather than having to shadow someone else all the time without being able to do things yourself, forcing you into the background. That erm well I like that a lot less.”* |
| Q5 | *“It’s teaching you lessons all the time, things to improve or to do differently next time…In that sense, it helps you to know what you’re doing and what you’ll do again next time. When you’re getting negative feedback, you’re not going to incorporate that into your professional attitude. But you will do so the other way around: when you look back on something you did really well or when you were friendly or erm eager, you’ll show that more often. It’s a learning process.”* |
| Q6 | *“And so you’re under a lot of pressure, with so many EPAs to obtain. It’s overstepping the mark, I think, actually you just want to get some feedback, which is very instructive and appropriate, but now there are so many EPAs that you worry about their number. That’s a bit of a shame, I think.”* |
| Q7 | *“I don’t fill it in myself because experience taught me that if I fill in the box myself, specialists just tick it off and move on without adding anything. I actually appreciate their feedback as input, so if I don’t fill anything in, they need to come up with something. A bit childish perhaps, but I leave a blank.”* |
| Q8 | *“You often get oral feedback while you haven’t really done any of the EPAs. So you do get feedback but you can’t tick your EPA form. Without knowing what EPA box to tick, I did actually get useful feedback on my professionalism.”* |
| Q9 | *“It’s different in each hospital, I noticed. In X they knew very well how it works, but in Y and in Z they were not so well informed; they would ask me what the mean was and I had to explain it to them.”* |
| Q10 | *“It’s different for each doctor: some will go through the entire physical examination with me, and some will simply credit what I’ve seen and found, and accept it.”* |
| Q11 | *“I thought it was useful for someone to monitor my development; in the intermediate review talk, she told me what points needed attention. At the end, she saw the improvements I’d made throughout the process, so yes. I also think it’s useful to be getting occasional feedback from someone else. I happened to have two supervisors, so that was great, the other one noticed different things that he thought were important. But I valued that there was someone who saw the whole thing.”* |
| Q12 | *“I prefer different people, I guess. In psychiatry I was supervised by an assistant physician for four weeks, and well she just knew that I was supposed to attain everything, all my targets. So she didn’t mind filling in the same stuff every time and gave me literally the same feedback over and over again. Well, that wasn’t very useful, you know.”* |
| Q13 | *“ Someone wrote a column in Medisch Contact, last week or something, saying that you’re actually starting all over again with every new post, every fortnight. And I thought to myself well yes, that’s actually true.”* |
| Q14 | *“I felt four weeks was long enough. Two weeks is a bit on the short side. You do need about a week and a half just to get used to things.”* |
| Q15 | *“When you’ve attained a particular EPA, you’re thinking ‘Wow, I’ve mastered it, and I can do this every time now’, something like that. In any case I think it helps to build your self-confidence, so you feel you can do it, and so I can actually be a doctor sometime.”* |
| Q16 | *“Yes bit of both, mainly how I feel about it, but when someone says ‘Well done, I’ll let you do it on your own next time’, well, that does help.”* |
| Q17 | *“No it happens as you go along. When you’re first seeing a patient in A&E, you’re thinking, oh dear, I just hope I’ll be fine. But when you’re really pleased with how something went, well, that ‘excellent’ on the feedback form is just a side issue. Or when you’ve made a diagnosis, for instance, and you’re told afterwards by the assistant physician that the neurologist thought the same, you don’t really need feedback, you already know it yourself.”* |
| Q18 | *“You do get more and more responsibilities but not on the basis of the EPAs you’ve attained. So there’s ongoing assessment of what they expect from you at that level and at that moment. In surgery, they just expect you to be able to do such and such. No one ever checks your EPAs except the final assessor or the intermediate assessor.”* |
| Q19 | *“I think that when the whole system has been properly established, it will help to have proper chats with doctors about what are your points for improvement and what’s going well.”* |
| Q20 | *“It’s very valuable that, through EPAs, all the feedback is documented in your portfolio, so you can show another supervisor what you have done before.”* |
